# Associations of breed, herd structure, and grazing system with the reproductive performance of Nguni and Boran cattle in South Africa

**DOI:** 10.3389/fvets.2026.1940048

**Published:** 2026-10-07

**Authors:** Mhlangabezi Slayi, Siphe Zantsi, Zuko Mpisana

**Affiliations:** 1 Department of Animal and Pasture Sciences, University of Fort Hare, Alice, South Africa; 2 Economic Analysis Unit, Agricultural Research Council (ARC), Pretoria, South Africa

**Keywords:** Boran, cow fertility, indigenous cattle, Nguni, pasture-based systems

## Abstract

Reproductive efficiency remains a major constraint limiting the productivity and sustainability of communal cattle production systems in sub-Saharan Africa. Although breed adaptation, herd structure, and grazing management influence cattle productivity, their combined effects on reproductive performance remain poorly understood. This study investigated the effects of breed adaptation, herd structure, and grazing system on the reproductive performance of Nguni and Boran cattle managed under communal rangelands in the Eastern Cape Province of South Africa. A total of 80 clinically healthy multiparous cows (40 Nguni and 40 Boran) were observed within a 2 × 2 × 2 factorial field design comprising two breeds, two herd structures evaluated as homogeneous (single-breed) or heterogeneous (mixed-breed) groups, and two grazing systems (cattle-only and mixed-species grazing). Reproductive traits, body weight, body condition score (BCS), and calf performance were monitored over a 12-month breeding cycle and analysed using mixed-effects models in R, with the herd/farm set as the primary experimental unit to reflect the herd-level application of treatments. Breed, herd structure, and grazing system were significantly associated with reproductive performance (*p* < 0.05). Nguni cattle achieved higher pregnancy (91.3%), conception (86.5%), calving (88.9%), and calf survival (96.2%) rates than Boran cattle. Heterogeneous herds and mixed-species grazing improved reproductive efficiency, body condition, and growth performance. Integrating adaptive indigenous breeds with improved grazing management can enhance reproductive performance and livestock productivity under communal rangelands.

## 1 Introduction

Livestock production remains one of the most important agricultural activities supporting food security, rural livelihoods, and economic development in sub-Saharan Africa (1, 2, 40–42). In South Africa, communal livestock systems contribute substantially to household income, nutritional security, cultural heritage, and socio-economic resilience, particularly among resource-constrained rural communities. However, the productivity of communal cattle herds remains constrained by several interacting factors, including poor grazing management, seasonal feed shortages, climate variability, disease challenges, and low reproductive efficiency (3, 4, 43–45). Reproductive performance is one of the most important determinants of herd productivity because it directly influences calf output, replacement rates, genetic improvement, and overall farm profitability (5, 6). Consequently, improving reproductive efficiency has become a key objective for achieving sustainable and climate-resilient livestock production systems (7, 8). Climate change has intensified the challenges facing communal livestock production through increasing temperatures, prolonged droughts, declining forage availability, and greater variability in pasture quality (9, 10). These environmental stressors reduce body condition, delay conception, increase embryonic losses, and ultimately lower calving rates and herd productivity (11, 27, 33, 34).

Among the management factors influencing reproductive performance, breed adaptation has received considerable attention. Indigenous cattle breeds have evolved under challenging environmental conditions characterized by nutritional limitations, disease pressure, and climatic variability, enabling them to develop physiological and behavioral adaptations that improve survival and productivity under low-input systems (12, 13). In southern Africa, Nguni cattle represent one of the most important indigenous breeds because of their tolerance to heat stress, endemic diseases and parasites, fluctuating feed resources, and harsh environmental conditions (14, 49). The Nguni is a small-framed breed highly localized to the region, known for its exceptional tick tolerance and high fertility under minimal management. Conversely, Boran cattle, although recognized for their superior growth potential, beef conformation, and an origin in East Africa that provides excellent metabolic efficiency during severe dry spells, possess higher dry-matter maintenance requirements that may limit reproductive performance under nutritionally constrained communal grazing environments (8, 15, 16). Both breeds leverage distinct adaptive mechanisms to sustain reproductive cycles in sub-optimal subtropical environments without extensive dietary inputs. Understanding how these breeds respond under different grazing management strategies is critical for optimizing communal livestock production.

Herd structure represents another important component of grazing management. In communal production systems, cattle are frequently managed either as homogeneous herds consisting of animals of a single breed or as heterogeneous herds containing different breeds managed together (2, 35, 36). Social hierarchy can substantially influence grazing behavior, movement patterns, and nutritional status. Heterogeneous herds may promote behavioral complementarity through differences in grazing preferences and forage selection, potentially improving pasture utilization and reducing direct competition among animals (17, 18). Similarly, mixed-species grazing has emerged as a promising strategy for improving rangeland productivity. Grazing cattle together with sheep and goats promotes complementary utilization of vegetation because different species consume different plant components (19–21). Although numerous studies have evaluated breed performance, grazing behavior, or mixed-species grazing independently, relatively few have investigated how breed, herd structure, and grazing system interact to influence reproductive performance under communal rangeland conditions. Most available studies have been conducted under commercial production systems, leaving an important knowledge gap regarding communal livestock systems where uncontrolled grazing and variable herd compositions interact. Therefore, the objective of this study was to determine the association of breed (Nguni and Boran), herd structure (homogeneous and heterogeneous herds), and grazing system (cattle-only and mixed-species grazing) on the reproductive performance of cattle managed under communal rangelands in the Eastern Cape Province of South Africa.

## 2 Materials and methods

### 2.1 Study area

The study was conducted across 20 communal livestock farms located within the Raymond Mhlaba Local Municipality, Eastern Cape Province, South Africa (32°47′S, 26°50′E), as systematically mapped out in Figure 1. The municipality is situated within a semi-arid agroecological zone characterized by communal livestock production, where cattle, sheep, and goats share natural rangelands under continuous grazing systems. Annual rainfall ranges from approximately 500 to 850 mm, with most precipitation occurring during the summer months (October to March). Mean annual temperatures range between 12 °C and 27 °C. The localized communal vegetation is dominated by mixed grassland and savanna species, including *Themeda triandra, Panicum maximum, Eragrostis curvula, Cynodon dactylon, Acacia karroo*, and Vachellia species, which provide both grazing and browsing resources throughout the year (22). The targeted communal grazing area directly borders the major town hub of Middledrift, representing a baseline cattle population managed comprehensively under continuous-use pastoral frameworks.

**Figure 1:**
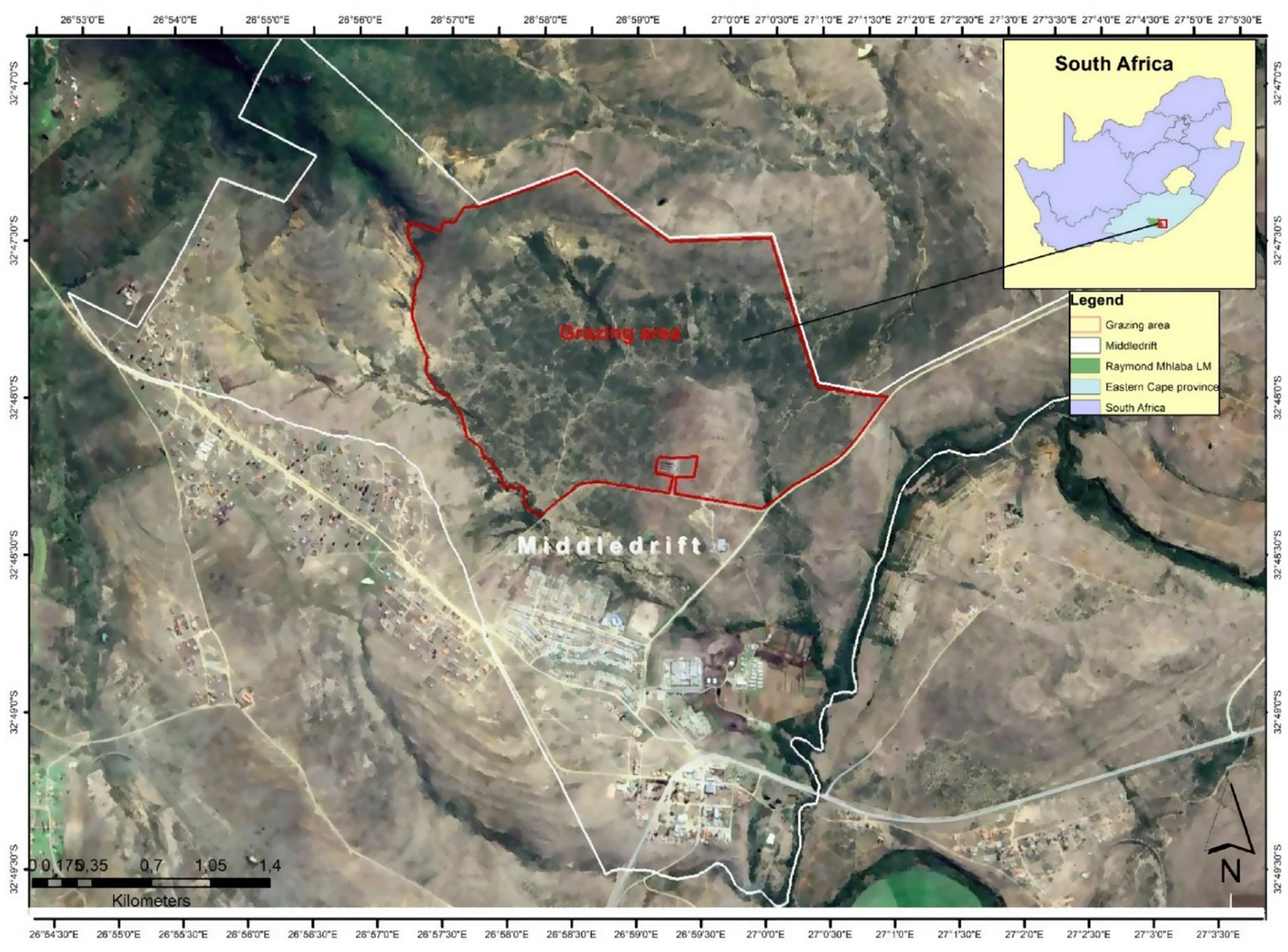
Map showing the location of the study site.

### 2.2 Ethical approval

Ethical approval for the study was obtained from the University of Fort Hare Animal Research Ethics Committee (Approval No.: MAP021SSLA01). All animal handling and experimental procedures complied with the South African National Standard for the Care and Use of Animals for Scientific Purposes. Written informed consent was obtained from all participating communal livestock farmers before commencement of the study.

### 2.3 Experimental animals and selection criteria

A total of 80 multiparous cows comprising 40 Nguni and 40 Boran cattle were selected from 20 participating communal livestock farms. The cows ranged strictly from 4 to 6 years of age to minimize the confounding effects of age on reproductive performance, thereby excluding highly variable heifers (under 2 years) or senescent cows (over 6 years). Parity was between 2 and 4, and the average initial body weight was 400 ± 25 kg. To ensure reproducibility, initial Body Condition Score (BCS) was assessed during the pre-breeding phase by two independent, trained scorers using a standardized 1 to 5 scoring system (where 1 = emaciated and 5 = obese) (50). Only cows maintaining a baseline BCS between 2.5 and 3.5 were selected to eliminate nutritional extremes from confounding the reproductive outcomes. Prior to selection, a comprehensive clinical examination was conducted by an animal health technician to rule out visible disorders, lameness, or parasitic infestation. To manage subclinical or asymptomatic conditions, baseline diagnostic screening was performed; all selected animals tested negative for brucellosis and bovine tuberculosis, and received routine vaccinations and prophylactic treatment for internal and external parasites according to provincial veterinary recommendations before the start of the experiment.

### 2.4 Experimental design and treatment allocation

The experiment was conducted over a 12-month period, encompassing one complete breeding and calving cycle. A 2 × 2 × 2 factorial field design was employed, with breed (Nguni and Boran), herd structure (homogeneous and heterogeneous), and grazing system (cattle-only and mixed-species grazing) serving as the main explanatory factors. Homogeneous herds consisted of animals from a single breed, whereas heterogeneous herds comprised both Nguni and Boran cattle managed together. The grazing system treatments included cattle-only grazing and mixed-species grazing. Because herd composition and grazing strategies in communal systems are determined collectively at the herd level rather than by individual animal manipulation, the management configurations were defined at the herd/farm level. A total of 20 communal farmers participated, with each farmer contributing a specific herd that conformed to one of the eight treatment combinations. The 80 selected cows were distributed across these 20 farms based on their baseline breed and management systems. Each treatment combination comprised exactly 10 cows across the participating farms, maintaining a balanced design. The distribution of farmers, treatment combinations, and animals per herd is detailed in Table 1.

**Table 1:** Treatment allocation and distribution of cows across the 20 participating communal farms.

Treatment	Breed	Herd structure	Grazing system	Number of farmers	Total cows (n)	Cows per farm
T1	Nguni	Homogeneous	Cattle only	3	10	3, 3, 4
T2	Nguni	Homogeneous	Mixed grazing	2	10	5, 5
T3	Nguni	Heterogeneous	Cattle only	2	10	5, 5
T4	Nguni	Heterogeneous	Mixed grazing	3	10	3, 3, 4
T5	Boran	Homogeneous	Cattle only	3	10	3, 3, 4
T6	Boran	Homogeneous	Mixed grazing	2	10	5, 5
T7	Boran	Heterogeneous	Cattle only	2	10	5, 5
T8	Boran	Heterogeneous	Mixed grazing	3	10	3, 3, 4

### 2.5 Grazing and reproductive management

All treatment groups grazed natural communal rangelands during daylight hours under extensive management. Stocking rates were maintained at approximately 6–8 ha per Large Stock Unit (LSU) depending on seasonal forage availability. Mixed-species grazing groups included cattle, sheep, and/or goats grazing simultaneously within the same herds at an average socio-spatial ratio of 1:4 (small ruminants to cattle). Water was available ad libitum, while mineral lick supplementation was provided uniformly across all farms. No concentrate supplementation was provided. Natural mating was practiced throughout the breeding season using fertile breeding bulls that had successfully passed a breeding soundness evaluation conducted according to the guidelines of the Society for Theriogenology (23). Bulls were introduced into each herd at a strict bull-to-cow ratio maintained between 1:25 and 1:30 across all groups. Pregnancy diagnosis was performed 60 days after the completion of the breeding season by an experienced veterinarian using transrectal ultrasonography (24).

### 2.6 Animal and productivity measurements

Body weight was measured monthly using a calibrated electronic livestock scale before morning grazing. Average daily gain (ADG) was calculated as the final body weight minus the initial body weight divided by the experimental period. Birth weight was recorded within 24 h after calving using a digital livestock scale. Weaning weights were recorded between 190 and 220 days of age, and all raw weights were mathematically adjusted to a uniform 205-day weaning weight using standard beef cattle performance testing guidelines to ensure accuracy.

### 2.7 Behavioral observations

Behavioral observations were conducted twice monthly throughout the 12-month experimental period using the focal animal sampling technique (25, 26). Each observation session lasted 8 h (08:00–16:00), during which trained observers recorded behavioral activities at 10-min intervals. The traits evaluated included grazing (active consumption of grasses), browsing (consumption of leaves, shrubs, and woody vegetation), walking (locomotion associated with forage searching), resting (standing or lying inactive), rumination, drinking, social interactions, and agonistic behavior (butting, chasing, or displacement) (27, 28). Behavioral activities were expressed as the percentage of the total observation time for each animal.

### 2.8 Statistical analysis

All raw data were verified in Microsoft Excel before being imported into R statistical software (Version 4.3.2; R Core Team, Vienna, Austria) for analysis. Data were screened for outliers and checked for normality using the Shapiro–Wilk test and homogeneity of variances using Levene’s test. Percentage data were arcsine-transformed where necessary to satisfy ANOVA assumptions; however, untransformed means are presented for ease of interpretation. Because management configurations, herd structures, and grazing systems were applied directly at the herd level across the participating communal farms, the herd/farm was designated as the primary experimental unit to avoid pseudo replication, while individual cow measurements were treated as observational subunits within herds. Continuous variables were analysed using a linear mixed-effects model (LMM) via the lme4 package:

$$\begin{array}{l} Y_{\text{ijkl}}=\mu +B_{i}+H_{j}+G_{k}+{\left(\mathrm{BH}\right)}_{\mathrm{ij}}+{\left(\mathrm{BG}\right)}_{\mathrm{ik}}+{\left(\mathrm{HG}\right)}_{\mathrm{jk}}\\ +{\left(\mathrm{BHG}\right)}_{\mathrm{ijk}}+F_{m}(H_{j}G_{k})+e_{\text{ijkl}} \end{array}$$

where: $Y_{\text{ijkl}}$ = observed response variable; *μ* = overall mean; = fixed effect of breed (*i* = 1, 2); = fixed effect of herd structure (*j* = 1, 2); *Gk* = fixed effect of grazing system (*k* = 1, 2); interaction terms represent two- and three-way interactions; *Fm* (*HjGK*) = random effect of the m-th Farm/Herd nested within herd structure × grazing system to account for intra-farm environmental clustering; and = residual error. Pregnancy, conception, and calving rates were analysed using generalized linear mixed models (GLMM) with a binomial distribution and logit link function, incorporating the same fixed and random effects structure. Treatment means were separated using Tukey’s Honestly Significant Difference (HSD) test, with statistical significance set at *p* < 0.05. Exact *p*-values are reported for all fixed effects and interaction terms to maximize transparency.

## 3 Results

### 3.1 Initial characteristics and baseline comparability

The initial age, body weight, body condition score (BCS), and parity of the experimental cows are presented in Table 2. Initial age, body weight, BCS, and parity were similar between Nguni and Boran cows (Table 2). However, because herd structure and grazing system were pre-existing farm-level configurations rather than randomly assigned treatments, the available study summary does not provide independent baseline tests for these two factors. Therefore, the present results do not claim baseline equivalence across herd structure or grazing system; the restricted eligibility criteria and balanced treatment allocation reduced, but did not eliminate, potential baseline imbalance.

**Table 2:** Initial body weight, body condition score (BCS), age, and parity of Nguni and Boran cows before commencement of the study (Mean ± SE).

Variable	Nguni (*n* = 40)	Boran (*n* = 40)	*p*-value
Age (years)	4.8 ± 0.2	4.9 ± 0.3	0.721
Body weight (kg)	398.6 ± 5.7	405.4 ± 6.1	0.384
Body condition score (1–5)	2.95 ± 0.08	2.91 ± 0.07	0.642
Parity	2.6 ± 0.2	2.7 ± 0.2	0.587

### 3.2 Reproductive traits and performance

The effects of breed, herd structure, and grazing system on specific reproductive traits are presented in Table 3. Breed significantly influenced all traits (*p* < 0.05), with Nguni cattle achieving greater pregnancy, conception, calving, calf survival, and weaning rates than Boran cattle. Herd structure and grazing system also showed significant associations with pregnancy, conception, and calving outcomes. Separate fixed-effect *p*-values are provided in Table 3 for each factor where available.

**Table 3:** Effect of breed, herd structure, and grazing system on reproductive performance parameters of indigenous cows.

Variable	Nguni	Boran	Homogeneous	Heterogeneous	Cattle only	Mixed grazing	SEM	Breed *p*-value	Herd structure *p*-value	Grazing system *p*-value
Pregnancy rate (%)	91.3^a^	80.2^b^	81.7^y^	89.8^x^	82.4^x^	88.9^x^	2.4	<0.001	0.011	0.018
Conception rate (%)	86.5^a^	75.4^b^	77.8^y^	84.6^x^	78.5^y^	84.2^x^	2.1	0.003	0.016	0.024
Calving rate (%)	88.9^a^	77.6^b^	79.3^y^	87.1^x^	80.2^y^	86.8^x^	2.3	0.002	0.014	0.028
Calf survival (%)	96.2^a^	91.4^b^	92.6	95.1	92.9	94.8	1.4	0.002	—	—
Weaning rate (%)	84.7^a^	73.5^b^	75.8	82.4	76.3	82.0	2.6	0.005	—	—

Means within a row under breed with different superscripts (a, b) differ significantly (*p* < 0.05). ^a,b^Means within a row under breed with different superscripts differ significantly (*p* < 0.05). ^x,y^Means within a row under herd structure or grazing system with different superscripts differ significantly (*p* < 0.05). SEM, standard error of the mean.

### 3.3 Fertility characteristics

Maternal breed exerted a prominent effect on fertility traits (Table 4). Nguni cattle required significantly fewer days to conception (54.3 ± 2.1 days) than Boran cattle (67.8 ± 2.7 days; *p* < 0.001). Nguni cows also required significantly fewer services per conception and maintained shorter calving intervals than Boran cows (*p* < 0.01). Gestation length did not differ significantly between the breeds (*p* = 0.217).

**Table 4:** Fertility characteristics of Nguni and Boran cows managed under communal rangelands (Mean ± SE).

Variable	Nguni	Boran	SEM	*p*-value
Days to conception	54.3 ± 2.1^b^	67.8 ± 2.7^a^	1.8	<0.001
Services per conception	1.36 ± 0.08^b^	1.74 ± 0.11^a^	0.05	0.004
Calving interval (days)	378 ± 6^b^	397 ± 8^a^	4.3	0.008
Gestation length (days)	283.7 ± 1.2	285.3 ± 1.4	0.9	0.217

^a,b^Means within a row with different superscripts differ significantly (*p* < 0.05). SEM, standard error of the mean.

### 3.4 Cow growth and body condition changes

Growth performance and body condition shifts during the breeding cycle are presented in Table 5. Breed and herd structure significantly influenced final body weight, weight change, average daily gain (ADG), and final body condition score (*p* < 0.05). Nguni cattle attained greater weight change and higher final BCS than Boran cattle. Concurrently, cattle maintained in heterogeneous herds outperformed those in homogeneous herds, recording significantly greater body weight gain, ADG, and final BCS values (*p* < 0.05).

**Table 5:** Growth performance and body condition changes of cows during the breeding season.

Variable	Nguni	Boran	Homogeneous	Heterogeneous	SEM	*p*-value
Final body weight (kg)	432.6ᵃ	427.4ᵇ	424.8ʸ	435.2ˣ	5.2	0.016
Weight change (kg)	34.0ᵃ	22.0ᵇ	23.8ʸ	32.4ˣ	2.4	<0.001
Average daily gain (kg/day)	0.38ᵃ	0.24ᵇ	0.26ʸ	0.36ˣ	0.02	<0.001
Final BCS (1–5)	3.38ᵃ	3.05ᵇ	3.09ʸ	3.34ˣ	0.07	0.002

ᵃ,ᵇMeans within a row under breed with different superscripts differ significantly (*p* < 0.05).ˣ^,^ʸMeans within a row under herd structure with different superscripts differ significantly (*p* < 0.05). SEM, standard error of the mean; BCS, body condition score.

### 3.5 Calf growth and pre-weaning performance

The effects of maternal breed on calf growth parameters are summarized in Table 6. Calves born to Boran cows had greater birth weights than those born to Nguni cows (*p* = 0.011). However, Nguni calves achieved significantly heavier adjusted 205-day weaning weights and higher pre-weaning ADG values than Boran calves (*p* < 0.05).

**Table 6:** Birth and pre-weaning growth performance of calves born from experimental cows (Mean ± SE).

Variable	Nguni calves	Boran calves	SEM	*p*-value
Birth weight (kg)	28.6 ± 0.5ᵇ	31.2 ± 0.6ᵃ	0.4	0.011
Adjusted 205-d weaning weight (kg)	182.5 ± 3.7ᵃ	176.3 ± 4.0ᵇ	2.6	0.039
Pre-weaning ADG (kg/day)	0.86 ± 0.03ᵃ	0.81 ± 0.03ᵇ	0.02	0.044

^a,b^Means within a row with different superscripts differ significantly (*p* < 0.05). SEM, standard error of the mean; ADG, average daily gain.

### 3.6 Fixed-effects analysis of selected primary outcomes

The fixed effects and associated interaction terms for selected primary reproductive and growth outcomes are detailed in Table 7. Breed, herd structure, and grazing system showed significant main effects for the outcomes reported in the table (*p* < 0.05). The table is intentionally restricted to selected primary outcomes and is not presented as a complete ANOVA for every response variable reported elsewhere in the manuscript. Significant breed × herd structure interactions were observed for pregnancy rate (*p* = 0.047), conception rate (*p* = 0.041), days to conception (*p* = 0.049), and ADG (*p* = 0.034), revealing that the impact of changing from homogeneous to heterogeneous groups differed between Nguni and Boran cattle. Breed × grazing system interactions significantly altered calving rate (*p* = 0.038) and ADG (*p* = 0.026). No significant three-way interactions were identified (*p* > 0.05).

**Table 7:** Fixed-effects analysis of selected primary reproductive and growth outcomes.

Source of variation	df	Pregnancy rate	Conception rate	Calving rate	Days to conception	Cow ADG
Breed (B)	1	<0.001	0.003	0.002	<0.001	<0.001
Herd structure (H)	1	0.011	0.016	0.014	0.021	<0.001
Grazing system (G)	1	0.018	0.024	0.028	0.039	0.002
Breed × Herd structure	1	0.047	0.041	0.184	0.049	0.034
Breed × Grazing system	1	0.115	0.092	0.038	0.074	0.026
Herd structure × Grazing system	1	0.324	0.411	0.295	0.512	0.187
Breed × Herd × Grazing	1	0.612	0.584	0.710	0.449	0.312

df = degrees of freedom; ADG, average daily gain. Interaction terms indicate whether the effect of one management factor depends on the level of another factor.

## 4 Discussion

The present study demonstrated that breed, herd structure, and grazing system significantly influenced the reproductive performance of cattle managed under communal rangeland conditions. Indigenous Nguni cattle consistently outperformed Boran cattle for pregnancy rate, conception rate, calving rate, calf survival, and weaning percentage, while heterogeneous herd structures and mixed-species grazing systems further enhanced reproductive efficiency. These findings demonstrate that reproductive performance under communal grazing systems is not solely determined by genetic potential but is strongly influenced by interactions between breed adaptation, social behavior, grazing ecology, and nutritional resource availability.

The superior reproductive performance of Nguni cattle is likely attributable to their long-term adaptation to southern African environments. Indigenous breeds have undergone natural selection under harsh climatic conditions characterized by seasonal feed shortages, high parasite pressure, and variable forage quality, resulting in lower maintenance nutrient requirements and greater resilience to nutritional stress. Nguni cattle maintain superior fertility under low-input production systems compared with larger-framed breeds (15, 16, 29). Conversely, Boran cattle exhibited comparatively lower reproductive indicators and longer periods to conception. This variation can be contextualized within the South African production framework: the Boran is a larger-framed breed with higher baseline metabolic maintenance costs. Under intensive tick challenges and seasonal forage scarcity typical of communal pastures, its higher energy demand compromises the proportion of dietary energy available for partitioning toward reproductive cycles, delaying post-partum estrus resumption (46–48).

Body condition is widely recognized as one of the strongest determinants of reproductive success because it directly influences ovarian activity and hormonal regulation. In the present study, Nguni cattle maintained higher body condition scores throughout the breeding season and consequently achieved higher conception and pregnancy rates, supporting the finding that maintaining adequate body reserves optimizes post-partum fertility (30, 31, 32, 37–39). Herd structure also exerted a significant influence on reproductive performance. Cattle maintained in heterogeneous herds recorded higher pregnancy, conception, and calving rates than those managed in homogeneous herds. This improvement may be explained by behavioral complementarity and social facilitation within mixed herds. Animals of different breeds often exhibit distinct grazing preferences and foraging strategies, resulting in more efficient exploitation of heterogeneous vegetation resources and reduced direct competition for forage (17, 18).

Mixed-species grazing systems further enhanced reproductive performance relative to cattle-only grazing systems. The biological explanation may lie in complementary resource utilization. Cattle primarily consume grasses, goats preferentially browse shrubs, and sheep utilize shorter grasses and herbaceous species. This dietary complementarity may reduce interspecific competition, suppress woody plant encroachment, and maintain greater total forage availability throughout the grazing season, thereby supporting the nutritional status of breeding cows (19–21). Behavioral observations were retained as descriptive measurements, but no individual-cow correlation analysis was used to infer reproductive relationships because cows were clustered within farms. Accordingly, behavioral variables are not interpreted as independent predictors of reproductive performance.

The observation that Boran calves were heavier at birth but Nguni calves achieved greater weaning weights provides additional insight into maternal breed adaptation. Larger birth weights in Boran calves reflect their greater mature size and genetic potential for fetal growth. However, the superior pre-weaning growth and adjusted weaning weights of Nguni calves suggest that maternal adaptation, milk production efficiency under extensive grazing conditions, and better maternal body condition may have enhanced calf growth after birth, matching patterns where moderate birth weights lower calving difficulties while efficient maternal performance supports superior calf survival under resource-limited conditions (15, 16). Beyond improving reproductive efficiency, these findings have important implications for sustainable livestock development and climate resilience. Improving conception rates, calving success, and calf survival directly increases herd productivity without increasing livestock numbers, thereby improving production efficiency while reducing grazing pressure per unit of output.

### 4.1 Limitations of the study

The field-based nature of this communal multi-farm study introduces several environmental and management variations that warrant cautious interpretation. First, individual bull effects, such as variations in libido, age, or physical fitness, could not be genetically or behaviorally standardized across the 20 participating farms, though baseline breeding soundness was verified. Second, the exact composition of the mixed-species grazing groups varied; some farms incorporated goats, others sheep, or a combination of both, which could introduce subtle differences in foraging competition and cattle behavior. Finally, animal distribution and herd sizes were dictated by the operational realities of communal setups rather than random allocation. In addition, farmers were not randomly assigned to herd structures or grazing systems; they were included because their pre-existing herds met the relevant management criteria. This creates a potential farmer self-selection bias, because management skill, resource access, motivation, prior experience, and other unmeasured characteristics may be associated with both the management configuration and livestock performance. The observed associations should therefore not be interpreted as definitive causal effects. Treating the farm/herd as the primary experimental unit and modeling farm-level clustering reduces pseudo replication but does not remove selection bias. The study also did not have sufficient summarized baseline information to formally test equivalence of age, body weight, BCS, and parity across herd-structure and grazing-system groups. Future studies should use prospective randomization or matched farm-level allocation, together with complete baseline measurements, to strengthen causal inference. Future prospective farm-level or controlled-station studies with randomized or matched allocation, standardized baseline measurements, and larger numbers of independent herds are recommended to isolate the biological, endocrine, and metabolic mechanisms linking grazing configurations to bovine reproductive physiology.

## 5 Conclusion

This study showed that breed, herd structure, and grazing system were associated with differences in reproductive performance of cattle managed under communal rangeland conditions. Nguni cattle achieved higher pregnancy, conception, calving, calf survival, and weaning rates than Boran cattle, consistent with the strong reproductive performance of indigenous cattle under semi-arid communal production systems. Furthermore, heterogeneous herd structures and mixed-species grazing systems were associated with improved reproductive efficiency and body condition, maintaining better body condition, and reducing competition for grazing resources. From a practical perspective, communal livestock farmers should prioritize well-adapted indigenous breeds such as Nguni cattle, while promoting heterogeneous herd structures and mixed-species grazing systems where resources permit. Agricultural extension officers have an important role in strengthening the capacity of communal farmers through continuous training on reproductive management, herd composition, and grazing planning. Policymakers should support programs that promote the conservation and utilization of indigenous cattle genetic resources and strengthen communal rangeland management institutions.

## Data Availability

The raw data supporting the conclusions of this article will be made available by the authors, without undue reservation.
